# Stochastic Methods for Inferring States of Cell Migration

**DOI:** 10.3389/fphys.2020.00822

**Published:** 2020-07-10

**Authors:** R. J. Allen, C. Welch, Neha Pankow, Klaus M. Hahn, Timothy C. Elston

**Affiliations:** ^1^Department of Pharmacology, University of North Carolina at Chapel Hill, Chapel Hill, NC, United States; ^2^Computational Medicine Program, University of North Carolina at Chapel Hill, Chapel Hill, NC, United States

**Keywords:** cell migration, stochastic modeling, RHOG, Rac1, biosenor, migration states

## Abstract

Cell migration refers to the ability of cells to translocate across a substrate or through a matrix. To achieve net movement requires spatiotemporal regulation of the actin cytoskeleton. Computational approaches are necessary to identify and quantify the regulatory mechanisms that generate directed cell movement. To address this need, we developed computational tools, based on stochastic modeling, to analyze time series data for the position of randomly migrating cells. Our approach allows parameters that characterize cell movement to be efficiently estimated from cell track data. We applied our methods to analyze the random migration of Mouse Embryonic Fibroblasts (MEFS) and HeLa cells. Our analysis revealed that MEFs exist in two distinct states of migration characterized by differences in cell speed and persistence, whereas HeLa cells only exhibit a single state. Further analysis revealed that the Rho-family GTPase RhoG plays a role in determining the properties of the two migratory states of MEFs. An important feature of our computational approach is that it provides a method for predicting the current migration state of an individual cell from time series data. Finally, we applied our computational methods to HeLa cells expressing a Rac1 biosensor. The Rac1 biosensor is known to perturb movement when expressed at overly high concentrations; at these expression levels the HeLa cells showed two migratory states, which correlated with differences in the spatial distribution of active Rac1.

## Introduction

The ability of cells to move is essential to many biological processes, such as tissue development, the immune response and wound healing (Franca-Koh et al., 2007; Petrie et al., 2009; Cain and Ridley, 2012). Anomalous cell migration plays a role in diseases, such as cancer and atherosclerosis (Cain and Ridley, 2012; Hall, 2009; Lemarié et al., 2010; Finney et al., 2017). During cell migration, intracellular signaling networks tightly control the spatiotemporal dynamics of the cytoskeleton. In particular, the Rho family of small GTPases has been implicated in membrane protrusion, adhesion, contraction and de-adhesion, all steps necessary for cell migration (Rottner et al., 1999; Jaffe and Hall, 2005; Goley and Welch, 2006; Ridley, 2006; Iden and Collard, 2008; Ladwein and Rottner, 2008). Rac1, the family member studied here, produces cell protrusions by interacting with effector proteins that modulate actin polymerization, including formins and Paks. A prevailing hypothesis is that Rac1 induces localized actin polymerization to trap random, thermal driven outward movements of the cell edge (Ridley, 2015; Marston et al., 2019; Schaks et al., 2019).

During random cell migration, in which cells do not experience directional environmental cues, cells move in a persistent manner, but with significant variability in their direction and speed. Therefore, methods for quantifying cell movement that take into account the stochastic nature of this phenomenon are needed. Previous studies have analyzed cell migration in terms of quantitative metrics such as the mean squared deviation in cell position, which can be linked to both speed and persistence (Othmer et al., 1988; Dimilla et al., 1992; Rosello et al., 2004; Dieterich et al., 2008). Additionally, it has been suggested that fractional diffusion models are required to accurately describe cell movement (Dieterich et al., 2008). We refer the reader to a recent review which describes these approaches and others (Svensson et al., 2018). We used stochastic modeling to develop tools for quantifying cell migration such that it can be characterized in terms of biologically relevant parameters. In our approach, the motion of cells is assumed to follow a 2D random walk with persistence. A related method that takes into account the probability of turning and contains a parameter related to persistence also has been applied to analyze random cell migration (Arrieumerlou and Meyer, 2005). An important distinction of our approach is that our model allows for the possibility of multiple states of migration, distinguished by differences in speed and persistence. This feature allowed us to determine that Mouse Embryonic Fibroblasts (MEFS) exist in two distinct states during random migration. Knock down of the Rho-GTPase RhoG suggests that this protein plays an important role in establishing the two states. We next demonstrated how our method allows the migration state of a cell to be predicted from time series data. Finally, we applied our method to examine the activation of Rac1, a GTPase known to be important in producing localized protrusions. Interestingly, we found that overexpressed, biosensor induced two states of migration in HeLa cells that correlated with different numbers of active Rac1 foci.

## Results

### Preliminary Analysis

**FIGURE 1:**
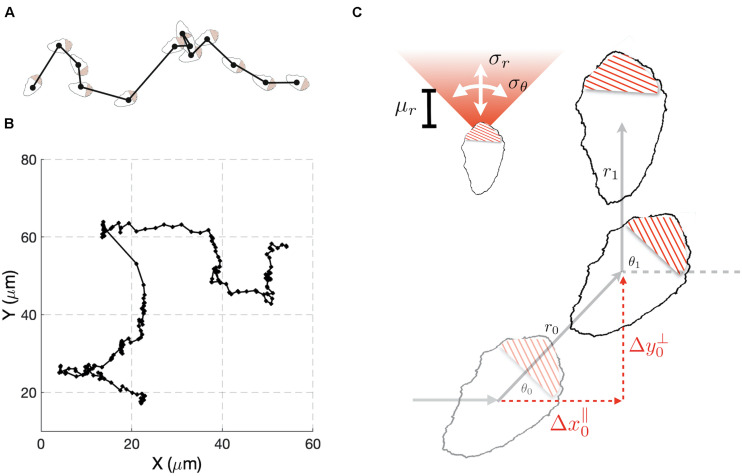
A stochastic model for cell migration. **(A)** Cell tracks are constructed by recording the geometric center of the cell over time. **(B)** Example track resulting from tracking the cell centroid at 5 min intervals (black dots). **(C)** A stochastic model of migration in which during each time interval, a cell moves a distance *r* through and angle θ with respect to the direction of the previous step. The random variable *r* is taken to be normally distributed with mean μ_*r*_ and variance $\sigma_{r}^{2}$. The angle θ is also normally distributed with mean zero and variance $\sigma_{\theta }^{2}$. To compare the model to experimental data we change variables to Δ*x*^∥^ and Δ*y*^⊥^, the directions parallel and perpendicular to previous step.

To develop our methods, we collected data sets that consisted of time series for the x and y coordinates of the cell centroids of randomly migrating MEF cells (Figures 1A,B). We chose this cell type because it shows persistent migration in the absence of directional cues. As an initial analysis of the data, we computed the average persistence of cell movement defined as *P* = < *cos*⁡(θ) >, where θ is the change in the direction of cell movement between measurements (Figure 1C) and the angular bracket denotes averaging over cell tracks. If θ is uniformly distributed, then the motion of the cell lacks persistence and *P* = 0. This behavior would be consistent with a pure random walk (diffusive motion). For values of *P* greater than zero, the movement of the cell shows persistence, with a value of 1 indicating motion in a straight line. Combining the cell tracks for individual cells, produced a value of *P* = 0.43. This value is consistent with cells that show persistent motion. We also generated histograms from the Δ*x* and Δ*y* displacements and empirically calculated cumulative density functions (Supplementary Figure S1, top left panel). These distributions were found to show slight deviations from a Gaussian distribution.

### A Stochastic Model for Cell Migration

Our preliminary cell track analysis led us to model cell movement as a 2D random walk with persistence (Figure 1C). In our model, for each time interval i, the distance, *r*_*i*_, traveled by a cell and the angle, θ_*i*_, through which the cell moves are considered random variables. The random variable *r*_*i*_ is taken to have a Gaussian distribution characterized by mean μ_*R*_, and variance $\sigma_{R}^{2}$. We allowed for negative values of *r*_*i*_ to account for the scenario in which a cell maintains its direction of polarization, but its centroid moves in a rearward direction. The directional angle θ_*i*_, is also taken to have a Gaussian distribution with variance $\sigma_{s}^{2}$, and centered on the value of the previous angle θ_*i–1*_. Small values of $\sigma_{s}^{2}$ correspond to highly persistent migration. For large values of $\sigma_{s}^{2}$ the new direction becomes uniformly distributed on the interval [−π, π] and the model represents a purely diffusive process.

It is not possible to tell from cell track data alone if changes in θ_*i*_ of magnitude greater than π/2 resulted from large deviations in orientation or negative *r*_*i*_. Thus, the probability distribution for these variables cannot be constructed unambiguously from the cell track data. To overcome this difficulty, we performed a change of variables from (*r*_*i*_,θ_*i*_) to ($\Delta \,x_{i}^{∥},\Delta \,y_{i}^{⊥}$), where these new variables correspond to changes in the centroid’s position during the *i*th time interval that are parallel and perpendicular to the direction of the previous step (Figure 1C). An important feature of the model is that analytical expressions for the probability density functions (pdfs) of $\Delta \,x_{i}^{∥}$ and $\Delta \,y_{i}^{⊥}$ can be found (Supplementary Information), allowing estimation of model parameters from experimental data to be performed in a computationally efficient manner, relative to the alternative of estimating probability density functions via repeated simulation of the stochastic model (Figure 1C). These co-ordinates explicitly handle the degeneracy in θ_*i*_ described above, because in these co-ordinates all possibilities that could have led to a given observation are considered. If cells show persistent motion, $\Delta \,x_{i}^{∥}$ has a positive mean value. Also, if there are no external cues in the experiments to define a preferred direction of motion, $\Delta \,y_{i}^{⊥}$ is symmetric about zero. Therefore, the distribution for $\Delta \,x_{i}^{∥}$ is more informative, and we use it to compare the experimental results with the model’s behavior. It is possible to simultaneously fit the $\Delta \,x_{i}^{∥}$ and $\Delta \,y_{i}^{⊥}$ distributions, but this comes at an increased computational cost. As a consistency check, after performing parameter estimation, we verify that the model accurately captures the $\Delta \,y_{i}^{⊥}$ distribution. If the model failed this consistency test, we could repeat the parameter estimation using both distributions. However, this was not required for any of the cases considered here.

We used a Monte Carlo method based on the Metropolis algorithm to perform parameter estimation. This was followed by local optimization algorithms to identify parameters associated with the global minimum error between the model and data (Supplementary Information). To test the accuracy and efficiency of this method, we benchmarked our approach using data generated from computational simulations of the stochastic model (Supplementary Figure S2). Having validated our computational methods, we next fit the model to the experimentally measured distributions. The model did not generate a good fit to experimental data for MEF cells (Supplementary Figure S3, dashed curve). In particular, we found that the model could not capture the second mode observed in the $\Delta \,x_{i}^{∥}$.

### A Multistate Model for Cell Migration

Further inspection of the MEF cell tracks suggested that individual cells might exist in different modes of migration, distinguished by differences in speed and persistence. We therefore expanded our model to allow for different states of migration. That is, we hypothesized that at any given time a migrating cell is in one of n states denoted by *S*_*i*_, with *i* ∈ {1…*n*}. Each state is characterized by the parameters $\mu_{r}^{i}$, $\sigma_{r}^{i}$, and $\sigma_{\theta }^{i}$. The additional parameters, α^*i*^, denoting the fraction of time spent in state *i*, are required to fully specify the model. Since ∑α^*i*^ = 1, in the two-state case the total number of parameters is seven. Note that if a two-state model is fit to data consisting of only a single state, then we expect our Monte Carlo method to produce parameter sets in which α^1^ takes on values of 0 or 1, or $\mu_{r}^{1}=\mu_{r}^{2}$, $\sigma_{r}^{1}=\sigma_{r}^{2}$, and $\sigma_{\theta }^{1}=\sigma_{\theta }^{2}$. The extended model is essentially a mixture model, which is itself a reduced hidden Markov model under the assumption that the probabilities of transitioning between states are independent and identically distributed. We again used simulated data to validate the accuracy and efficiency of our Monte Carlo method when multiple states are considered (Supplementary Figure S4).

**FIGURE 2:**
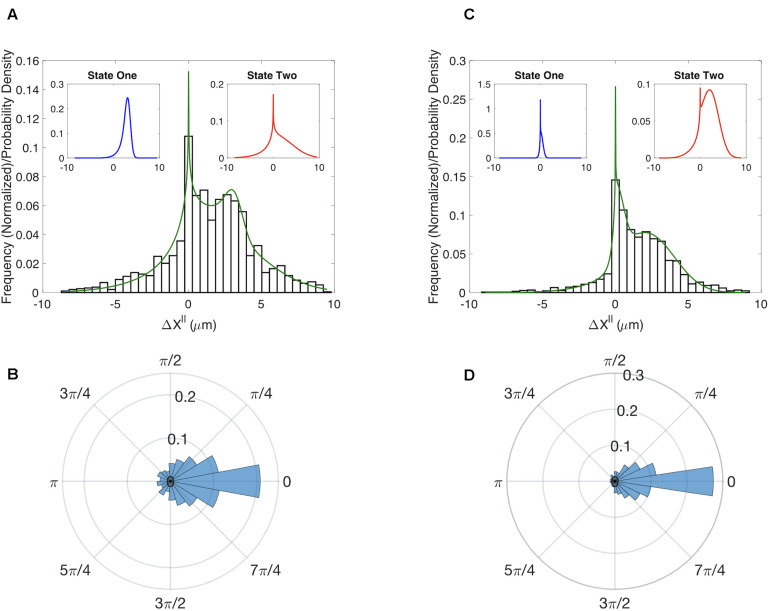
Results for the multistate model of migration. **(A)** Comparison of the experimentally determined distribution of Δ*x*^∥^ for WT MEF cells (histogram) to the results of a two-state model (green curve). Insets show the distributions for the predicted two states. **(B)** Experimentally determined distribution for the angle θ for WT MEF cells. **(C)** Same as **(A)** except for cells in which RhoG has been knocked down. **(D)** Same as B except for cells in which RhoG has been knocked down.

The multi-state model produced a good fit to the MEF $\Delta \,x_{i}^{∥}$ distribution (Figure 2A). To assess the accuracy of our parameter estimates we used confidence-interval profiling (Raue et al., 2009). To determine acceptable values for the sum of the squared errors (SSE) we boot-strapped the original datasets to assess plausible differences in our observed distributions should we repeat the experiments (Supplementary Information). The results of this analysis provide a measure of the confidence that should be placed on each estimated parameter value (Supplementary Figures S5A,B). Of particular interest is the parameter α which represents the fraction of time in each state. The best fits were achieved with α = 0.12. We confirmed that the model also captured the distributions for $\Delta \,y_{i}^{⊥}$ (Supplementary Figure S6A). The results of our analysis suggest that randomly migrating MEFs exist in one of two states. About 12% of time these cells are in a state with a well-defined characteristic step of ∼3 μm (State 1 – blue distribution in Figure 2A left inset) and an angular distribution with $\sigma_{\theta }^{1}=0.7$. In the second state, the step size is highly variable (State 2 – red distribution Figure 2A right inset) and the motion is less persistent $\sigma_{\theta }^{2}=1.3$. For completeness, we also show the distribution for the angle θ (Figure 2B).

### RhoG’s Role in Migration

It has long been appreciated that the canonical Rho-GTPases RhoA, Rac1, and Cdc42 play important roles in cell migration. However, the role of RhoG in migration is less well studied. To determine if RhoG plays a role in the random migration of MEFs, we generated time series data for cells in which this protein was knocked down. While the angular distributions for the WT and knockdown do not show clear differences (Figures 2B,D), the Δx_∥_ distributions indicate RhoG does effect migration (Figures 2A,C). Moreover, by fitting our simple two-state model we can quantify this effect and ascertain that the persistent state 1 in the MEF control has been converted to a state in which the cells do not show significant movement ($\mu_{r}^{1}=3.2\,\mu$m for the WT to $\mu_{r}^{1}=0.25\,\mu$m for the KD). State 2 seems to be preserved by the KD in the sense that the confidence intervals defining state 2 parameters are overlapping in the two cases (Supplementary Figure S5). A putative mechanism for how RhoG activation influences cell migration via recruitment of the DOCK180/ELMO complex (Katoh and Negishi, 2003; Katoh et al., 2006), which acts as GEF for Rac1. However, whether this is the key pathway in this process, and how it is organized spatio-temporally, is a direction of future research.

### Inferring States From Time Series Data

We next sought to develop computational tools that could be used to determine if the predicted states of migration correspond to subpopulations of cells with distinct phenotypes or if individual cells could transition between states. To test if individual cells change their migration state, we developed a method to infer migration states from individual cell tracks. Our approach uses a Bayesian prediction method based on the probability that a sequence of *k* successive steps arises from one of the identified states (see Methods for details). Before applying our state prediction method on the experimental data, we first validated the approach using synthetic data. To generate this data, we performed computational simulations of the stochastic model using the parameters estimated from the experimental data for MEF cells. With these values our state-prediction algorithm correctly identified the states more than 90% of the time, validating the approach (Supplementary Figure S7).

**FIGURE 3:**
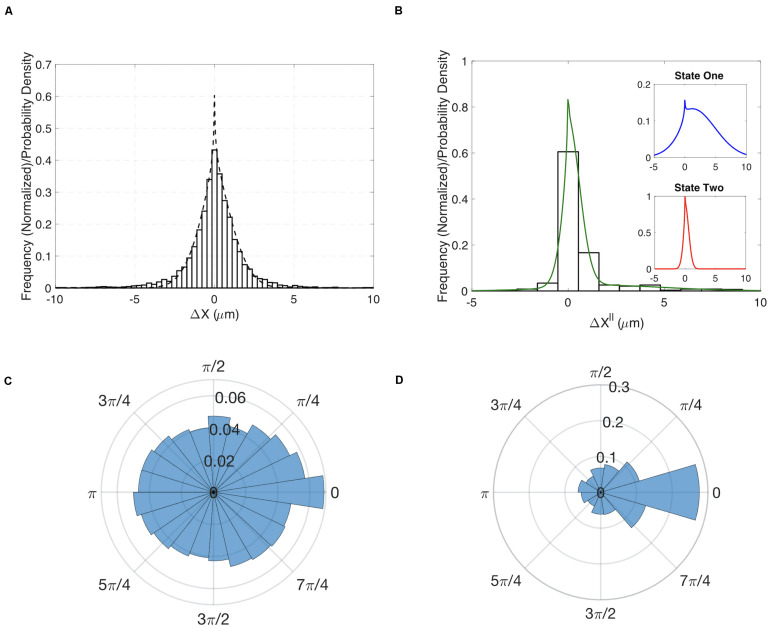
Results for HeLa cells with and without expression of a Rac1 biosensor. **(A)** Comparison of the experimentally determined distribution of Δ*x*^∥^ for HeLa cells not expressing the biosensor (histogram) to the results of a one-state model (dashed curve). Insets show the distributions for the predicted two states. **(B)** Experimentally determined distribution for the angle θ for HeLa cells not expressing the biosensor. **(C)** Comparison of the experimentally determined distribution of Δ*x*^∥^ for HeLa cells expressing Rac1 biosensor (histogram) to the results of a two-state model (green curve). Insets show the distributions for the predicted two states. **(D)** Experimentally determined distribution for the angle θ for HeLa cells expressing biosensor.

**FIGURE 4:**
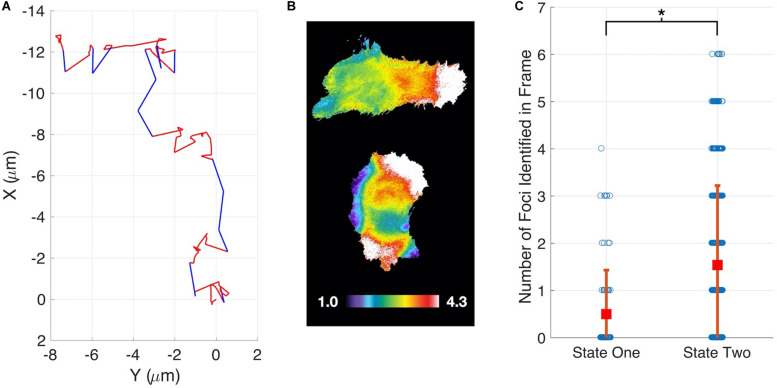
State prediction for HeLa Cells expressing a Rac1 biosensor. **(A)** Example cell track showing switching between a fast-persistent state (blue) and slower-less persistent state (red). **(B)** When in the fast-persistent state (state 1, upper panel) cells have fewer active Rac1 foci as compared to the slower-less persistent state (state 2, lower panel). The upper cell is undergoing persistent motion to the right. The color scale indicates the ratiometric readout of Rac activation, normalized so the lowest 5% of the cell = 1. **(C)** Quantification of the number of foci in each state.

Having demonstrated our method’s ability to infer cell migration states from simulated track data and demonstrate a role for RhoG, we examined whether the different migration states could be correlated with molecular changes within cells. The Hahn lab has used HeLa cells to develop new biosensors and optogenetic probes. It is well established that these molecular tools must be used at controlled concentrations, below levels where they perturb cell movement (Kraynov et al., 2000; Machacek et al., 2009). Controls in earlier studies have shown that HeLa cells exhibit altered motility when the Rac1 biosensor is expressed at high levels. We decided to investigate if our stochastic modeling approach could quantify the effects of biosensor overexpression. We compared WT HeLa cells without biosensor expression to cells with high levels of Rac1 biosensor. Our analysis revealed that WT cells showed little directed motion and a single migration state was sufficient to capture the distributions of steps sizes (Figures 3A,B). In contrast, cells with highest levels of biosensor exhibited two states of migration (Figure 3C). In particular, two states were needed to capture the long tail of the distribution (see Supplementary Figure S9 for comparison of one-and two state results). In state 1 the cell moves in persistent manner, whereas in state 2 the cell is mostly stationary. To test if the predicted two states are correlated with differences in cell signaling, we ran our state prediction algorithm on the track data. Interestingly, our analysis predicted that individual cells randomly switch between the two states (Figure 4A), and qualitative observations indicated that the slow state showed multiple disperse Rac activation events at the edge of the cell, while the fast state showed a single Rac activation at the leading edge (see Supplementary Movies M1, M2). To quantify this observation, we identified and counted the number of foci of active Rac1 in each image and grouped these counts by the predicted state (see Supplementary Information for details), reasoning that random movement would require more cell protrusions distributed around the cell perimeter. Rac activation is known to be sufficient to generate cell protrusions (Wu et al., 2009; Wang et al., 2016). Cells predicted be in state 1, which corresponds to the fast-persistent state, had fewer Rac1 foci than those predicted to be in state 2, which show little net movement (Figure 4B). This observation is consistent with highly motile cells typically showing strong polarity.

## Discussion

We developed novel computational methods for analyzing the movement of randomly migrating cells. Our approach combines stochastic modeling with statistical inference methods to detect and quantify migratory phenotypes. Migrating cells have a biochemical, morphological, and structural orientation that persists as these cells move. Our model captures this ‘memory’ by conditioning the cell’s movement during the current time interval on its previous direction of motion. An important feature of our model is that analytic expressions for the probability densities for cell displacements parallel and perpendicular to the previous direction of motion can be found. This feature allows us to generate the probability density function for a given set of parameters rather than generating an approximation to this PDF via stochastic simulation of our migration model (Figure 1C). In most use cases the analytical PDF is computationally more efficient due to the high number of repeats required to estimate the PDF with suffcient accuracy. We have validated all our approaches using simulated data, and then applied the methodology to study randomly migrating MEF and HeLa cells.

Our modeling approach allows for multiple states of migration. This feature allowed us to demonstrate that migrating cells randomly transition between modes of movement. Crucial to the detection of these states is the quantification of parameter values and the associated confidence in those estimates. This process allowed us to be confident in the existence of two states of migration for MEF and HeLa cells over-expressing a Rac1 biosensor.

The identification of multiple states of migration for MEF cells led us to assess the role of RhoG in establishing these states. To do this we used siRNA to reduce RhoG expression. This perturbation suggests that RhoG plays a role in directed migration, because reducing RhoG eliminated net movement in the first predicted state and shortened the range of step sizes taken in the second state. We next developed a Bayesian approach to predict the current migration state of a cell from time series of the cell’s position. Using this method, we demonstrated that individual HeLa cells expressing a Rac1 biosensor switched between migratory states. Importantly, we were able to correlate these two states with differences in the distribution of Rac1 activity.

We believe that our methods provide useful tools for quantifying and characterizing cell migration. Our stochastic model characterizes cell migration using parameters with straightforward biological interpretations. Hence, application of this model can lead to biological insights not apparent in the data from visual inspection or simple quantitative measures. In this case, our analysis suggests a role of RhoG in allowing cells to change direction, which may play a role in the ability of randomly migrating cells to search their environment.

## Materials and Methods

### Computational Methods

The full code and analysis for this paper is available (Allen, 2020).

#### Coordinate Transformation

We modeled cell migration as a stochastic sequence of steps characterized by the step size *r*_*i*_ and directional angle θ_*i*_ (Figure 1). Since we assume *r*_*i*_ and θ_*i*_ to be realizations of independent random variables *R* and Θ the probability the cell moves (*r*,θ) is defined by

(1) $$f\,\left(r,\theta |\theta_{i-1}\right)=g_{R}\,\left(r\right).g_{\Theta }\,\left(\theta |\theta_{i-1}\right)$$

where *g*_*R*_(*r*) is the probability density function (pdf) for the step magnitude, which we take to have the normal distribution $𝒩\,\left(\mu_{r},\sigma_{r}^{2}\,\Delta \,t\right)$, and *g*_Θ_(θ|θ_*i*−1_) is the pdf generating the new orientation conditioned on the previous angle, which we take to have the normal distribution $𝒩\,\left(\theta_{i-1},\sigma_{\theta }^{2}\,\Delta \,t\right)$. The experimental data is collected in Cartesian coordinates *(X, Y)*. In principle we could transform the data into the coordinates *R* and Θ. However this transformation cannot be completed uniquely, because there is no way to distinguish a backward step in which the cell maintains its direction of polarity (θ_*i*_ = θ_*i*−1_) from one in which the front and back of the cell have reversed (θ_*i*_ = θ_*i*−1_ + (2*k* + 1)π). Furthermore, the value of θ_*i*_ cannot be determined if *r*_*i*_ = 0. For these reasons, we transform the model to the coordinates ($\Delta \,x_{i}^{∥},\Delta \,y_{i}^{⊥}$), where these new variables correspond to changes in the centroid’s position during the *i*th time interval that are parallel and perpendicular to the direction of the previous step.

To compare with the model the data needs to be manipulated to generate histograms for steps in the *x*^∥^ and *y*^⊥^ directions. For each sequential triplet of coordinates {(*x*_*i*−1_,*y*_*i*−1_),(*x*_*i*_,*y*_*i*_),(*x*_*i* + 1_,*y*_*i* + 1_)}, we rotate the steps as a rigid body about (*x*_*i*−1_,*y*_*i*−1_) by a four quadrant inverse tangent based on $\tan^{-1}\frac{y_{i}-y_{i-1}}{x_{i}-x_{i-1}}$. The result of this is that all steps are pre-orientated in a positive x-direction and initiated at (0,0), and can be plotted as histograms of step distance in the *x* and *y* direction: $\left(\Delta \,x_{i}^{∥},\Delta \,y_{i}^{⊥}\right)=\left(x_{i+1}^{\prime }-x_{i}^{\prime },y_{i+1}^{\prime }-y_{i}^{\prime }\right)$.

The pdf for Δ*x*^∥^ is:

(2) $$f_{X}\,\left(\Delta \,x^{∥}\right)=\int_{-1}^{1}\frac{f_{H}\,\left(\Delta \,x^{∥}/h,h\right)}{h}\,dh$$

where *h* = cos(θ), and,

(3) $$f_{H}\left(\Delta x^{∥}/h,h\right)=g_{r}\left(\Delta x^{∥}/h\right)\sum_{k}\frac{1}{{\left(1-h^{2}\right)}^{\frac{1}{2}}}\times \;\,\left(g_{\Theta }\,\left(\arccos\left(\text{h}\right)+2\,\pi \,k\right)+g_{\Theta }\,\left(-\arccos\left(\text{h}\right)+2\,\pi \,k\right)\right).$$

The expression for *f*_*Y*_(Δ*y*^⊥^) is similar, however now with *g*_Θ_(*arccos*⁡(h) + π/2 + 2π*k*) + *g*_Θ_(−*arccos*⁡(h) + π/2 + 2π*k*) in the summation term. A derivation of these results is presented in the Supplementary Information.

#### Parameter Estimation

Parameters were estimated by simulated annealing, which is a Monte Carlo method based on the Metropolis algorithm (24, 25). Initial choices of parameters generate an analytical solution (Eq. 2), which is scored against the experimental data $\left(\Delta \,x_{i}^{∥}\right)$ by the sum of least squared differences. At each step of the algorithm the parameters are updated by a small addition of Gaussian noise, if this update scores better than the current score then these parameters are accepted. If the score is higher, the parameter set is accepted with probability $e^{-\frac{\Delta \,s}{T}}$, where Δ*s* is the difference between the current and previous scores and *T* is the current temperature. Over the course of the fitting *T*, the temperature is reduced. This fixes the parameter choices into a local minimum. Here we choose a geometric cooling regime. Due to the stochastic nature of the simulation, and that there could be many local minima, it is necessary to run this fitting procedure multiple times. The best fit of this routine was then further refined using MATLABs fmincon routine, which was also used to assess the sensitivity of our fit to altering parameter values via confidence-interval profiling (Supplementary Figure S4, Supplementary Material for details).

The histograms were amalgamated from multiple cell tracks. For the case of two states, the pdf for Δ*x* becomes

$$f_{X}\,\left(\Delta \,x\right)=\alpha \,f_{X}^{1}\,\left(\Delta \,x\right)+\left(1-\alpha \right)\,f_{X}^{2}\,\left(\Delta \,x\right)$$

where α is the fraction of time spent in state 1 and the distributions $f_{X}^{1}\,\left(\Delta \,x\right)$ and $f_{X}^{2}\,\left(\Delta \,x\right)$ are parameterized by $\left(\mu_{r}^{1},\sigma_{r}^{1},\sigma_{\theta }^{1}\right)$ and $\left(\mu_{r}^{2},\sigma_{r}^{2},\sigma_{\theta }^{2}\right)$, respectively.

Parameter sets were identified by multiple simulated annealing runs, followed by local-optimization routines.

#### Validation of Methods

To validate the pdfs and the parameter estimation algorithm, we simulated cell tracks using the stochastic model (Figure 1). Cell tracks were generated using two states, each with distinct parameter sets. At each step a state was chosen at random with probability 0.5. As above, the simulated cell tracks were used to construct the distributions for $\Delta \,x_{i}^{∥}$ and $\Delta \,y_{i}^{⊥}$. We assumed model parameters were not known and used the Monte Carlo method to fit Eq. 2, modified to two states (see below) to the simulated data for $\Delta \,x_{i}^{∥}$. The Monte Carlo method quickly converged on the correct parameter values (Supplementary Figure S4), validating the analytical solution to the model and our fitting procedure. In theory we also could fit the pdf for $\Delta \,y_{i}^{⊥}$. However, the pdf for $\Delta \,y_{i}^{⊥}$ is symmetric, because there is no preferred direction of migration and therefore less informative than the distribution for $\Delta \,x_{i}^{∥}$. We found that we could maintain the accuracy of our parameter estimation while improving the computational cost by only considering the $\Delta \,x_{i}^{∥}$ distribution. As a consistency check, we always verify that the estimated parameters accurately reproduce the pdfs for $\Delta \,y_{i}^{⊥}$ (Supplementary Figure S6).

#### State Prediction

To identify which state a cell is in at a given time, we used Bayes’ theorem to invert the problem. That is, we calculate the probability that a cell is in state *S*_*i*_ given the experimental data. Note that in calculating this probability, we also get the false positive rate or p-value. To make a reliable prediction of *S*_*i*_ may require an n-step window, where n is odd, such that, {*x*_*i*−*n*/2_,…,*x*_*i*−1_,*x*_*i*_,*x*_*i* + 1_,…,*x*_*i* + *n*/2_}. Then:

$$P\,\left(s_{i}|X\right)=\frac{P\,\left(X|s_{i}\right)\,P\,\left(s_{i}\right)}{P\,\left(X|s_{i}\right)\,P\,\left(s_{i}\right)+P\,\left(X|s_{i}^{c}\right)\,P\,\left(s_{i}^{c}\right)}$$

where *P*(*X*|*s*_*i*_) is calculated from the model, and we take *P*(*s*_*i*_) = α. Windows of length one, three and five were tested. For the case presented here, we found that the window of length one produced results similar to the other two window lengths.

#### Foci Identification

Ratiometric images of the FRET based Rac1 biosensor were analyzed for localized regions of higher Rac1 activity near the periphery of the cell. We call these regions “foci”. We used custom application of the image processing toolbox in MATLAB to identify foci, which we define as contiguous regions within the cell that were simultaneously: (1) 60% above the average intensity of the cell, (2) greater than 100 pixels in area, and (3) contained at least one point within 5 pixels of the cell edge. The length of time (or number of frames) that a cell could be followed for varied. So, to not overweight any one cell, the number of image frames analyzed, *n*, was selected to maximize *n* × *m* where *m* is the number of cells with at least *n* images.

### Experimental Methods

#### Cell Culture and Transient Transfections

HeLa cells were maintained in Dulbecco’s modified Eagle’s Medium (DMEM) (Cellgro) supplemented with 10% fetal bovine serum (FBS) (HyClone), 100 U/mL penicillin and 100 μg/mL streptomycin (Cellgro) and 2 mM L-glutamine (Invitrogen) at 37°C and 5% CO_2_. All cDNA constructs were transfected into cells using FuGene6 (Roche) according to the manufacturer’s instructions. IA32 Mouse Embryonic Fibroblast (MEF) cells were maintained in Dulbecco’s modified Eagle’s Medium (DMEM) (Cellgro) supplemented with 10% fetal bovine serum (FBS) (HyClone) and 1× GlutaMAX (Thermo Fisher Scientific).

IA32MEFs were transfected with either RhoG siRNA (CAGGTTTACCTAAGAGGCCAA) or Allstars Negative Control siRNA (Qiagen, United States). 7.5 μL, 10 μM siRNA was added to 250 μL serum-free DMEM. 3 μL lipofectamine RNAimax was added to another 250 μL serum-free DMEM. After 5 min, the two solutions were mixed and incubated for 20 min, followed by dropwise addition to a 35 mm dish. Medium was changed after 24 h and cells were split as required for use in experiments 48–72 h post-transfection, when knock-down efficiency was maximal. Control siRNA cells were incubated with 5 μM CFDA green for 20 min in serum-free DMEM. CFDA-labeled control cells were mixed with unlabeled RhoG siRNA cells immediately prior to the experiment.

#### Live Cell Imaging

For live cell imaging, cells were plated on fibronectin-coated coverslips (10 μg/ml fibronectin) 4 h before imaging, then transferred to Ham’s F12-K imaging medium supplemented with 2% FBS and 15 mM HEPES. Live cell imaging was performed in a closed heated chamber (20/20 Bionomic).

For biosensor imaging, photobleach-corrected time-lapse image stacks were acquired for 18 h at 5 min intervals and processed as previously described (Pertz et al., 2006; Machacek et al., 2009; Hodgson et al., 2010). The following filter sets were used (Chroma Technology Corp.): CFP: D436/20, D470/40; FRET: D436/20, ET535/30; YFP: D500/20, ET535/30. Cells were illuminated with a 100 W Hg arc lamp through a 1.0 neutral density filter.

For RhoG siRNA experiments cell tracks were generated through 10× DIC imaging of cells plated as above, but using Ham’s F12K medium supplemented with 5% FBS. Images were acquired for at least 70 frames at 10 min intervals in a closed, heated chamber. This length of track was objectively identified as optimal by maximizing the total number of analyzed frames in the entire data set.

## Data Availability Statement

The raw data supporting the conclusions of this article will be made available by the authors, without undue reservation.

## Author Contributions

RA, TE, and KH devised the research plan and wrote the manuscript. RA performed the mathematical and computational calculations. CW and NP collected the data. All authors contributed to the article and approved the submitted version.

## Conflict of Interest

The authors declare that the research was conducted in the absence of any commercial or financial relationships that could be construed as a potential conflict of interest.

## Supplementary Material

FIGURE S1 Cumulative distribution functions (CDFs) for *x* and *y* step sizes compared to the CDFs for normal distributions.

FIGURE S2 Validation of analytical approach. Simulated data (histogram) is generated by simulating data (blue bars) stochastically with parameters (μ_*r*_,σ_*r*_,σ_θ_,σ_0_) = (0.1,0.1,0.5,π/5). Comparison with the analytical PDF (red) gives good agreement.

FIGURE S3 Fitting the model to MEF WT cells indicates that a one state model of migration cannot adequately capture key features of the data. Observed data (open bars), model fit (green).

FIGURE S4 Analytics and Fitting Validation. Simulated data (histogram) is generated by applying the model with parameters $\left(\mu_{r}^{1},\sigma_{r}^{1},\sigma_{\theta }^{1}\right)=\left(1,0.3,3\right),\left(\mu_{r}^{2},\sigma_{r}^{2},\sigma_{\theta }^{2}\right)=\left(5,1,0.5\right)$ and α = 0.5. Assuming the underlying parameters were unknown, we used simulated annealing to fit the analytical solution (green line). This validates our analytical solution and fitting procedure (we estimate $\left({\hat{\mu }}_{r}^{1},{\hat{\sigma }}_{r}^{1},{\hat{\sigma }}_{\theta }^{1}\right)=\left(1.02,0.30,2.72\right),\left({\hat{\mu }}_{r}^{2},{\hat{\sigma }}_{r}^{2},{\hat{\sigma }}_{\theta }^{2}\right)=\left(5.08,0.93,0.51\right)$, and (α = 0.50).

FIGURE S5 Parameter values and associated confidence intervals. For each condition, and each parameter, the best-fit is the minimum of the blue curve. Acceptable parameter values are those which can lead to a value below the threshold (red dots) when the other parameters are re-fit, highlighted by the red-dash lined. Note, that two states were clearly identified in both cases.

FIGURE S6 Model prediction versus the Δ*Y* distribution from the cases shown in Figure 2. **(A)** MEF WT **(B)** MEF RhoG KD. In **(A)** and **(B)**: Observed data (open bars), model fit (green) and individual pdfs for state one and state two (insets).

FIGURE S7 Testing the accuracy of predicting states using HeLa Rac1 model parameters. Data simulated stochastically, where we know the state a given step was generated from, is compared with our prediction of the state using Bayes Theorem. Overall, we are correct more than 90% of the time. However, steps truly in state one get occasionally mischaracterized are overlapping leading to an accuracy of around 70% in this case.

FIGURE S8 Bootstrapped Analysis of the Difference in Foci Between States. To ascertain if the difference we observed in the mean foci count in state one versus state two was meaningful or could have been observed by chance, we performed a boostrap analysis. For a given iteration of the bootstrap, we randomly assigned state one or state two to each image frame with probability α and 1−α, respectively. Then, for each iteration, calculated Δ mean foci as the difference in the mean number of foci in state one versus state two. Blue bars, bootstrapped distribution (50,000 iterations), dotted red line observed Δ mean foci.

FIGURE S9 Comparison of 1-state (dashed curves) and 2-state (green curves) model fits for all of the experimental results.

FIGURE S10 Overview of algorithm to estimate model parameters and confidence intervals.

FILES M1 M1 (slow_state_hela_rac1.mp4) and M2 (fast_state_hela_rac1.mp4). The predicted “fast state” of migration was correlated with fewer Rac1 foci, most frequently a single focus at the leading edge, and in the slow state multiple brief Rac1 activation foci appear.
