# Supplementary figures and images for: Stochastic Methods for Inferring States of Cell Migration
Source: Front Physiol. 2020 Jul 10;11:822. doi: 10.3389/fphys.2020.00822 (PMC7365915; doi:10.3389/fphys.2020.00822)

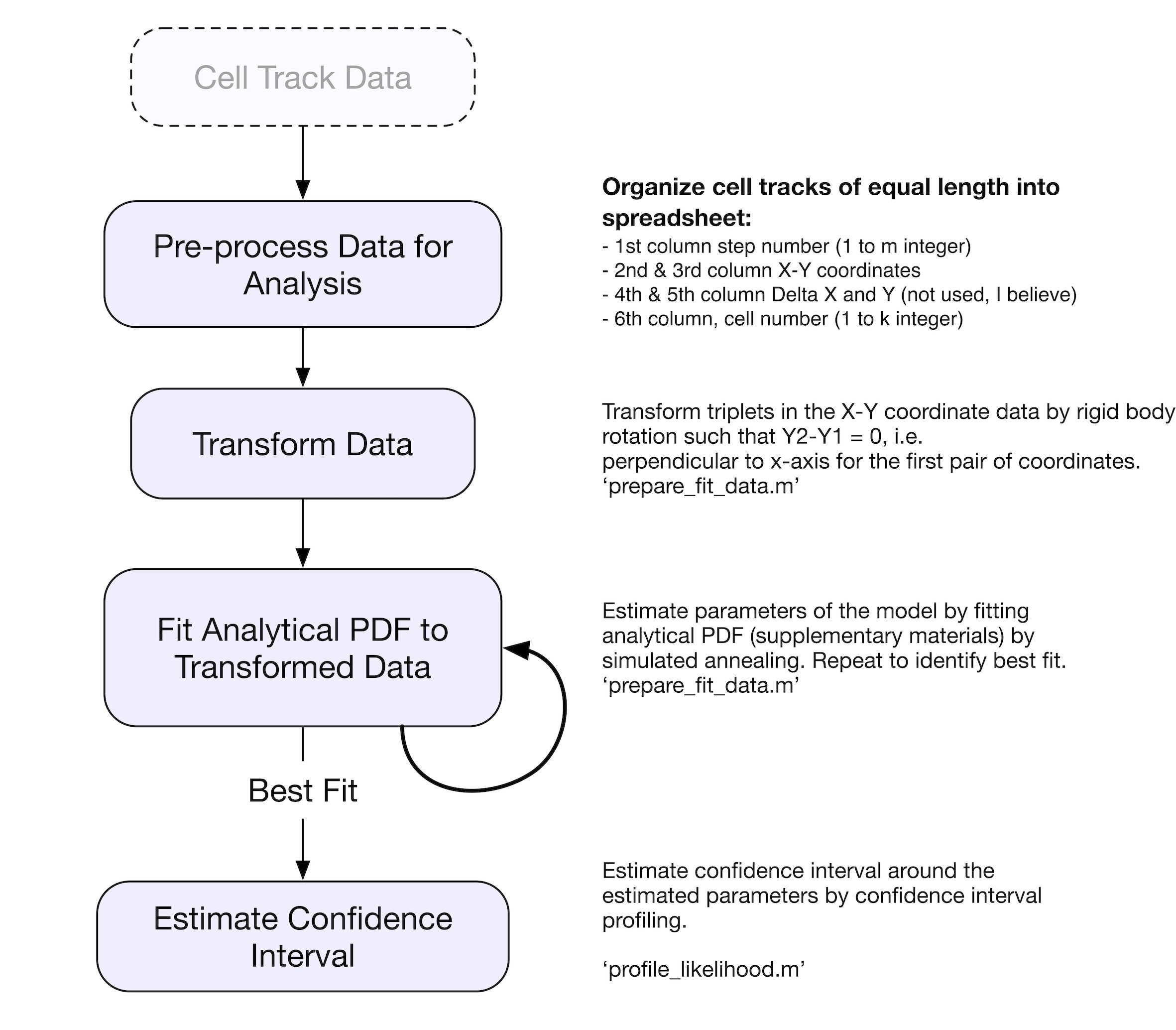

Supplement: FIGURE S1 — Cumulative distribution functions (CDFs) for x and y step sizes compared to the CDFs for normal distributions. [file Image_1.JPEG]
