# Supplementary material for: Stochastic Methods for Inferring States of Cell Migration
Source: Front Physiol. 2020 Jul 10;11:822. doi: 10.3389/fphys.2020.00822 (PMC7365915; doi:10.3389/fphys.2020.00822)
Supplement: FIGURE S2 — Validation of analytical approach. Simulated data (histogram) is generated by simulating data (blue bars) stochastically with parameters (μr,σr,σθ,σ0) = (0.1,0.1,0.5,π/5). Comparison with the analytical PDF (red) gives good agreement. [file Data_Sheet_2.PDF]

Sup Fig 1

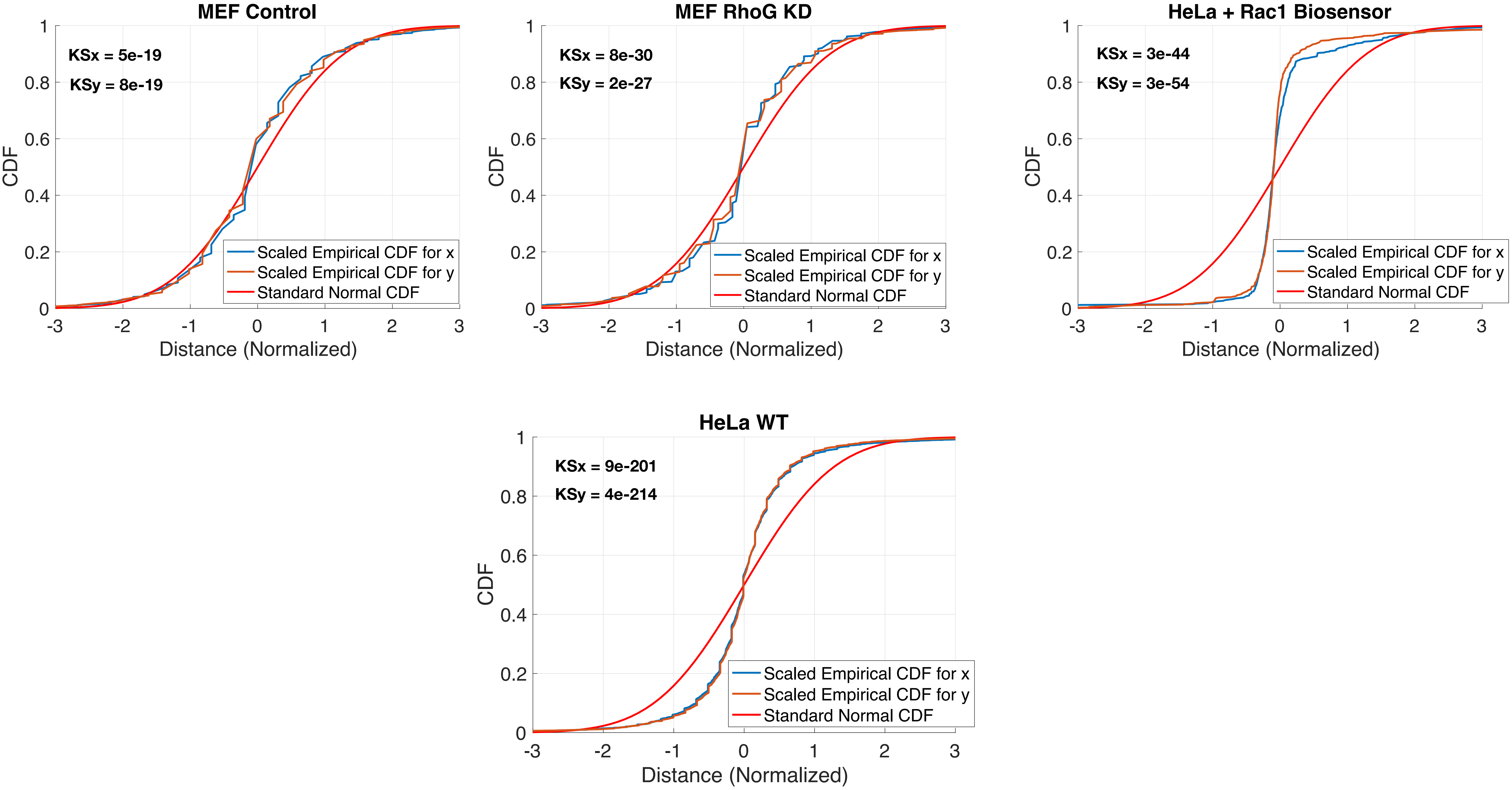
MEF Control
KSx = 5e-19
KSy = 8e-19
Scaled Empirical CDF for x
Scaled Empirical CDF for y
Standard Normal CDF
CDF
Distance (Normalized)
MEF RhoG KD
KSx = 8e-30
KSy = 2e-27
Scaled Empirical CDF for x
Scaled Empirical CDF for y
Standard Normal CDF
CDF
Distance (Normalized)
HeLa + Rac1 Biosensor
KSx = 3e-44
KSy = 3e-54
Scaled Empirical CDF for x
Scaled Empirical CDF for y
Standard Normal CDF
CDF
Distance (Normalized)
HeLa WT
KSx = 9e-201
KSy = 4e-214
Scaled Empirical CDF for x
Scaled Empirical CDF for y
Standard Normal CDF
CDF
Distance (Normalized)

Sup. Fig. 2

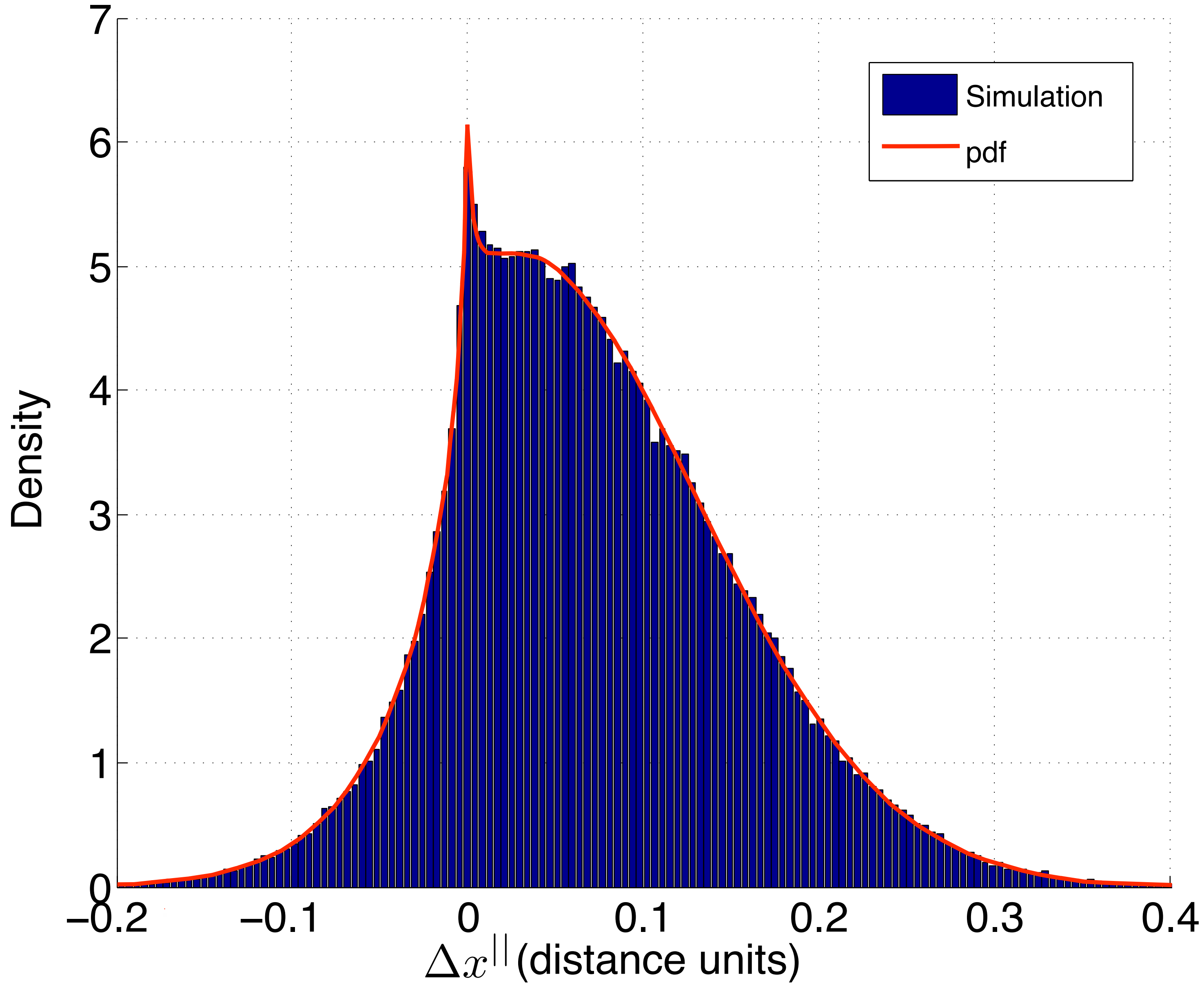

Simulation
pdf
Density
7
6
5
4
3
2
1
0
−0.2
−0.1
0
0.1
0.2
0.3
0.4
$\Delta x^{||}$ (distance units)

Sup. Fig. 3

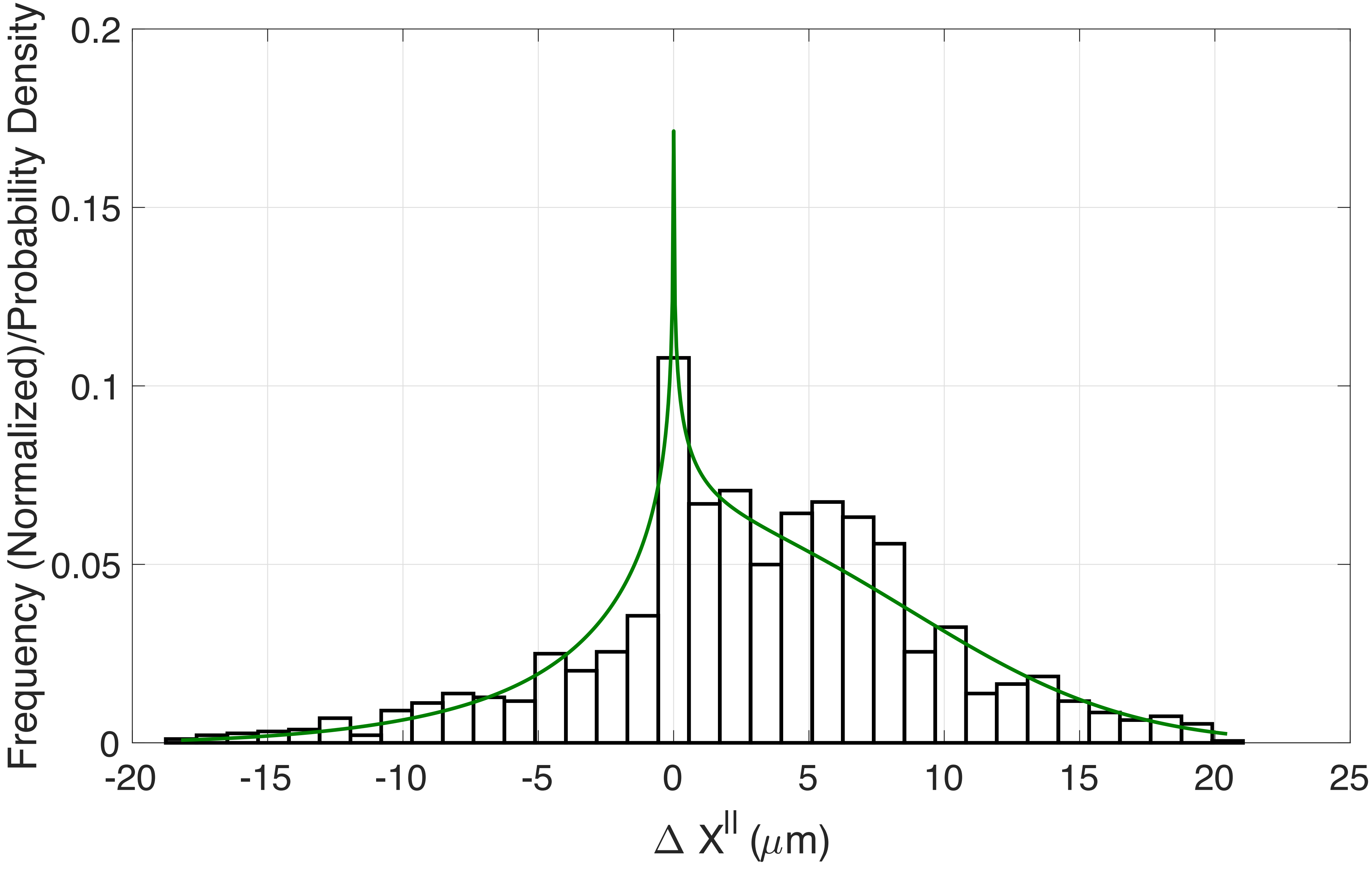

Frequency (Normalized)/Probability Density
0.2
0.15
0.1
0.05
0
-20
-15
-10
-5
0
5
10
15
20
25
Δ X^{ll} (μm)

Sup. Fig. 4

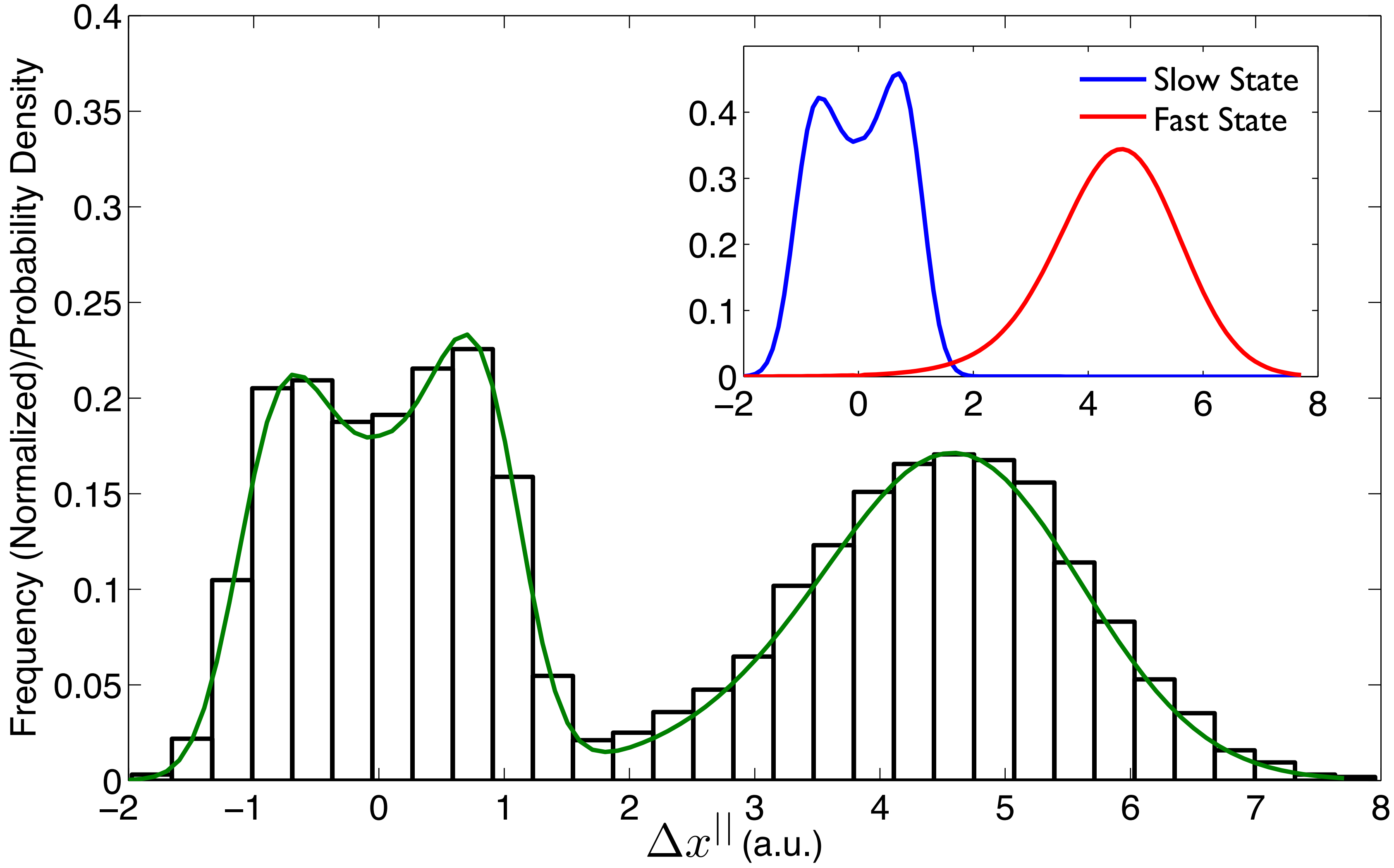

Slow State
Fast State
Frequency (Normalized)/Probability Density
$\Delta x^{||}$ (a.u.)

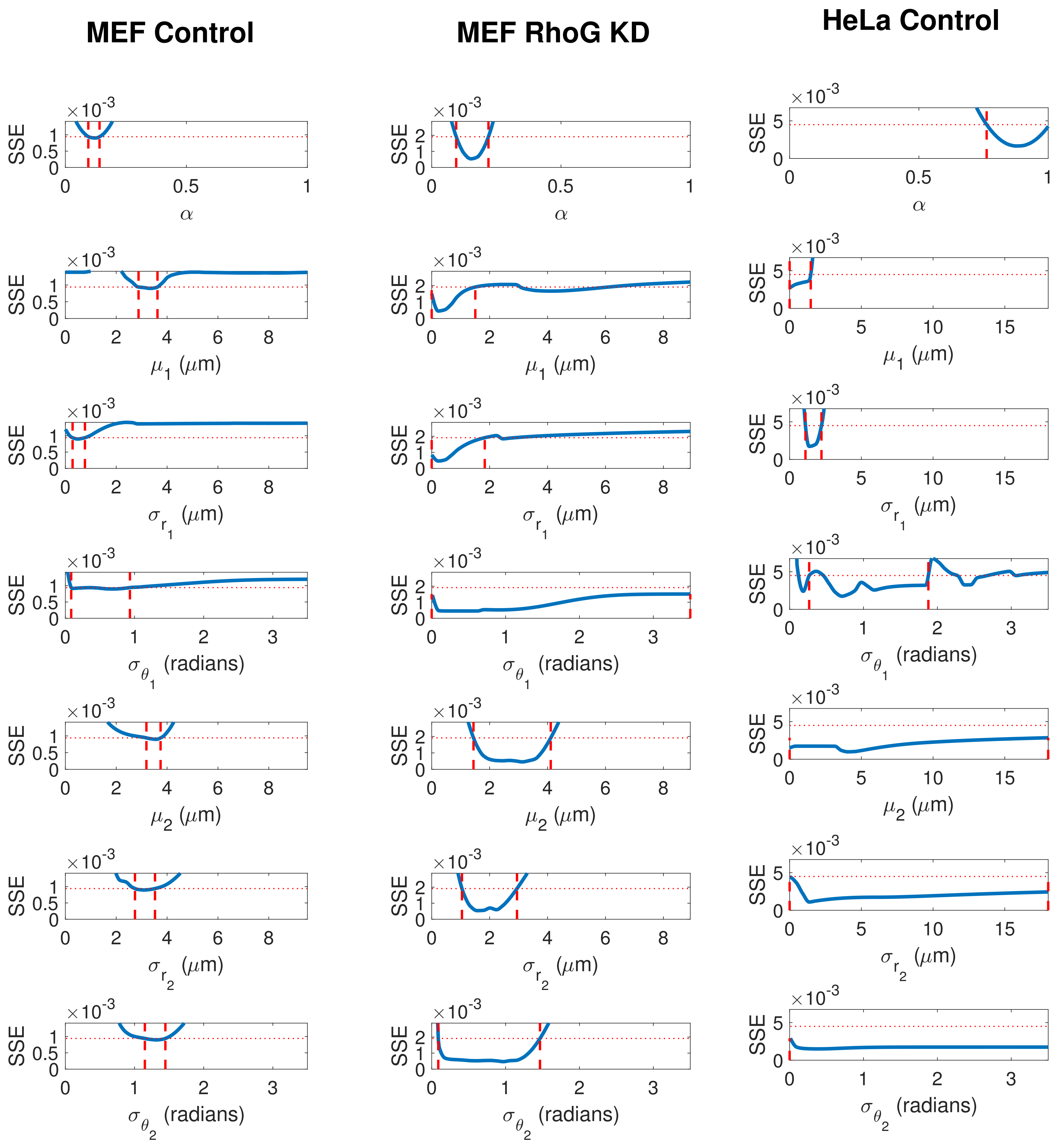
MEF Control
MEF RhoG KD
HeLa Control
SSE
α
μ_1 (μm)
σ_{r_1} (μm)
σ_{θ_1} (radians)
μ_2 (μm)
σ_{r_2} (μm)
σ_{θ_2} (radians)
×10^-3

Sup. Figure 5

Sup. Fig 6

A

B

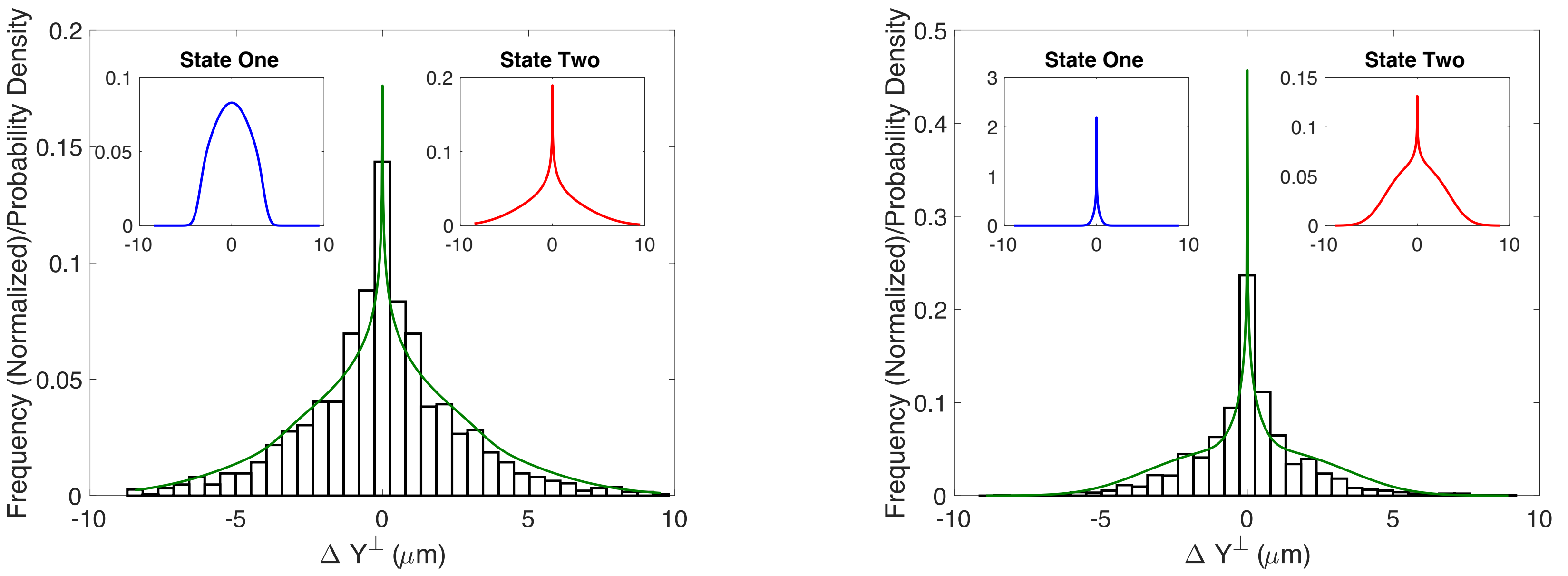

MEF Control

MEF RhoG KD

Sup. Fig 7

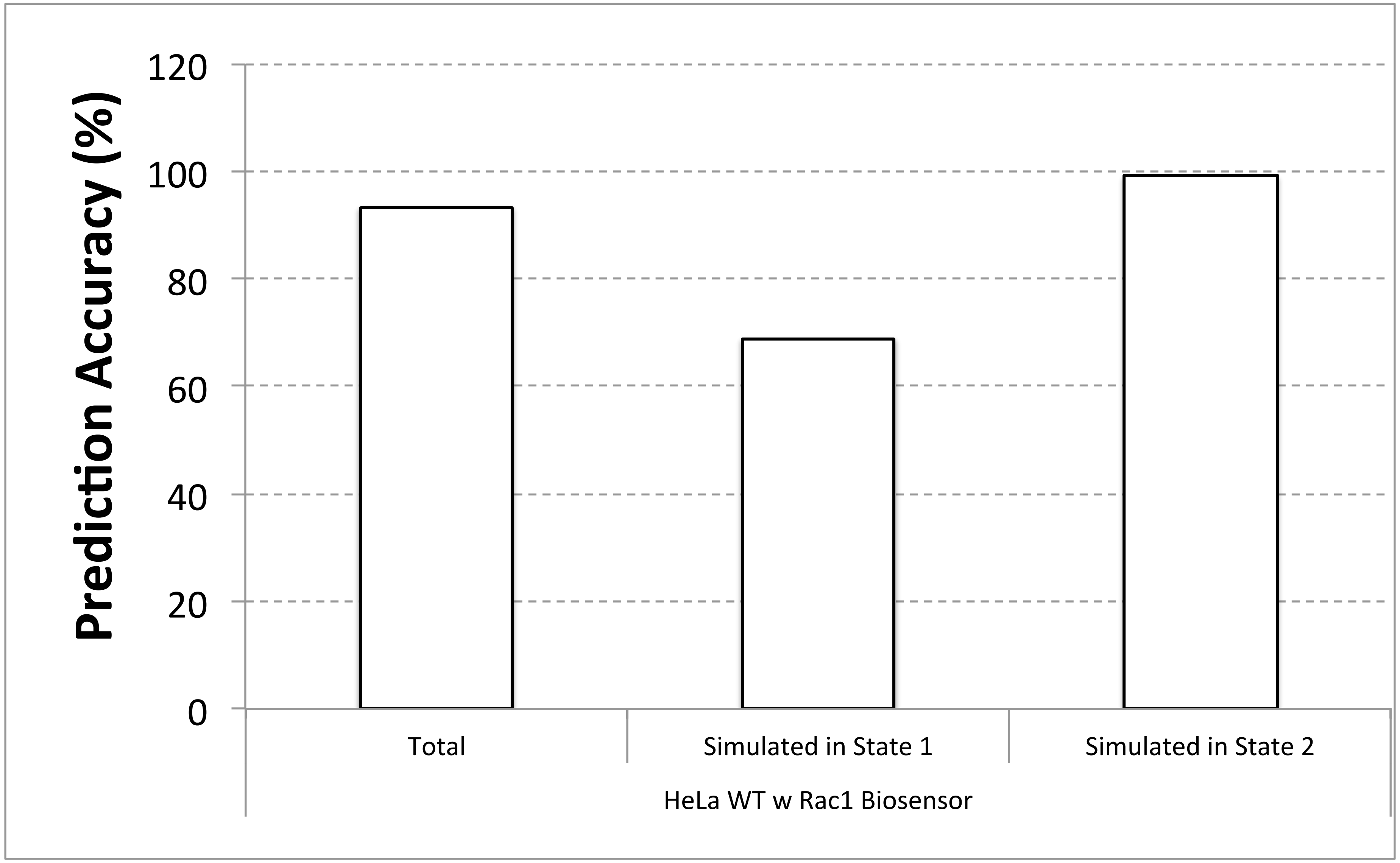
Prediction Accuracy (%)
120
100
80
60
40
20
0
Total
Simulated in State 1
Simulated in State 2
HeLa WT w Rac1 Biosensor

Sup. Fig. 8

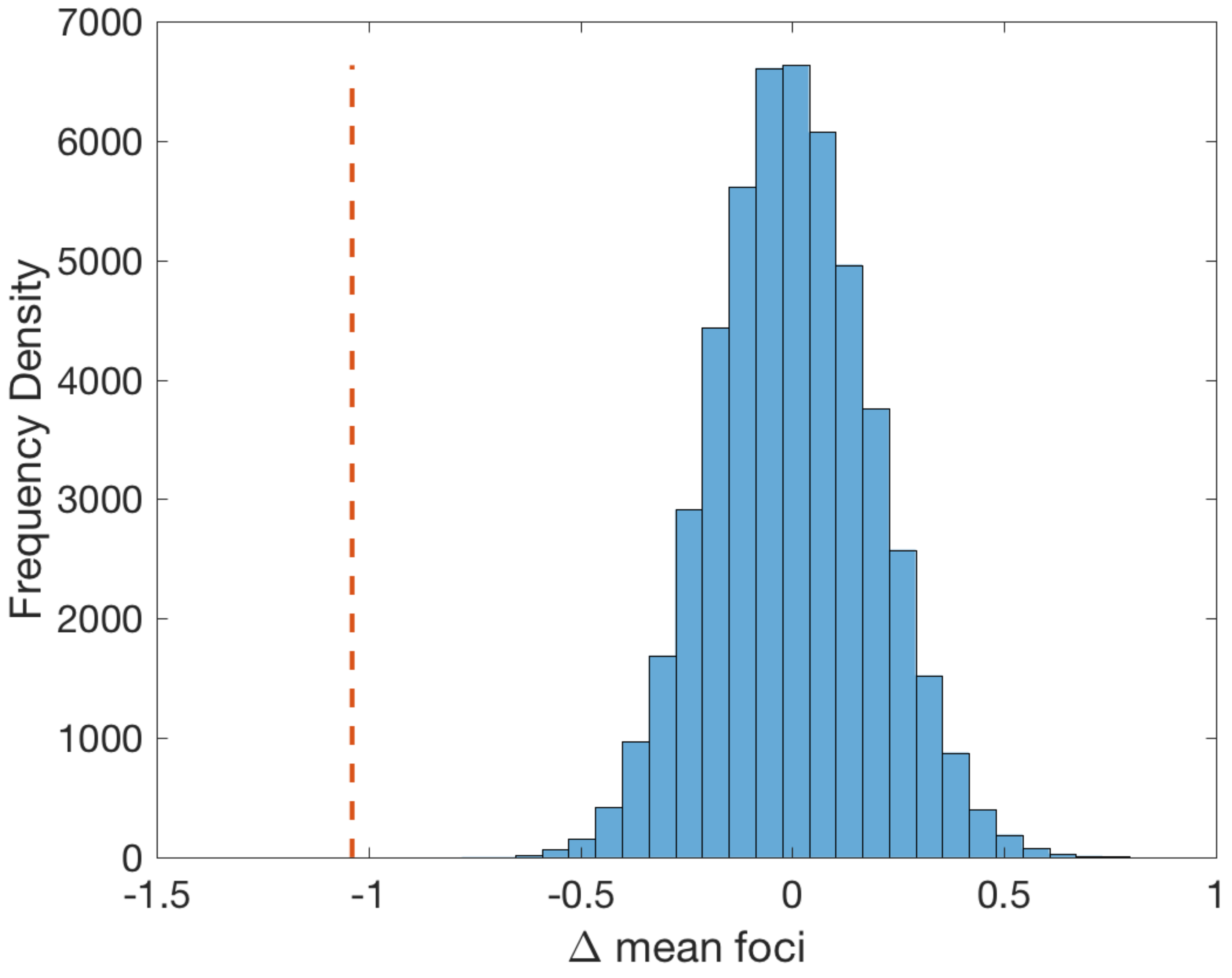

7000
6000
5000
4000
3000
2000
1000
0
Frequency Density
-1.5
-1
-0.5
0
0.5
1
Δ mean foci

Sup. Fig. 9

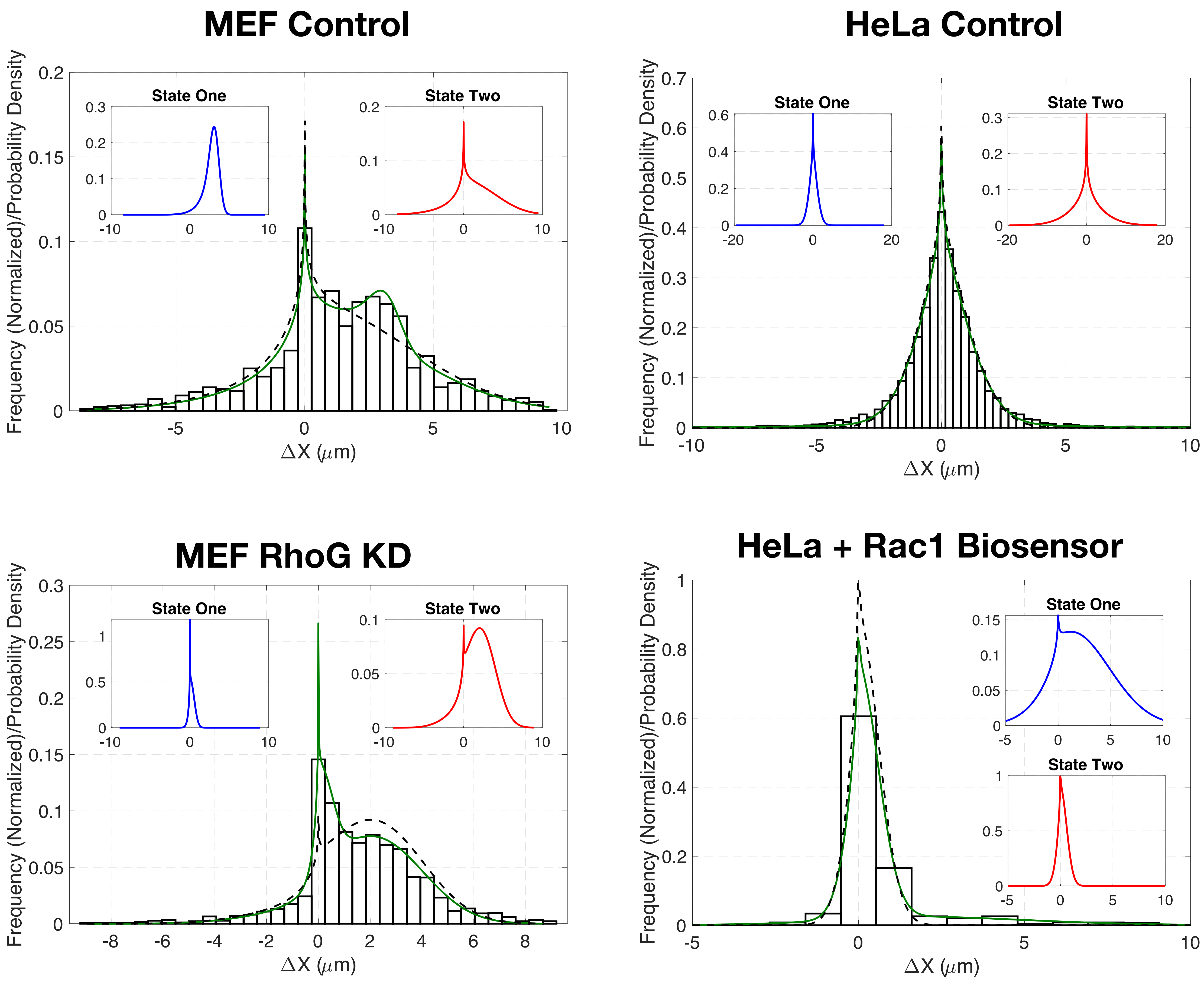
MEF Control
State One
State Two
Frequency (Normalized)/Probability Density
ΔX (μm)
HeLa Control
State One
State Two
Frequency (Normalized)/Probability Density
ΔX (μm)
MEF RhoG KD
State One
State Two
Frequency (Normalized)/Probability Density
ΔX (μm)
HeLa + Rac1 Biosensor
State One
State Two
Frequency (Normalized)/Probability Density
ΔX (μm)
