# Supplementary material for: Stochastic Methods for Inferring States of Cell Migration
Source: Front Physiol. 2020 Jul 10;11:822. doi: 10.3389/fphys.2020.00822 (PMC7365915; doi:10.3389/fphys.2020.00822)
Supplement: FILES M1 — M1 (slow_state_hela_rac1.mp4) and M2 (fast_state_hela_rac1.mp4). The predicted “fast state” of migration was correlated with fewer Rac1 foci, most frequently a single focus at the leading edge, and in the slow state multiple brief Rac1 activation foci appear. [file Data_Sheet_1.PDF]

Supplementary Materials

1 Analytical Solution of Cell Migration Model

Important to our analysis of cell migration is that our model of cell migration has an analytical expression which can be compared to experimental data. We can generate cell tracks stochastically using, (see figure 1):

$$\begin{aligned} R_t &\sim N(\mu t, \sigma_r^2 t) \\ \Theta_t &\sim N(\theta_0, \sigma_\theta^2 t) \end{aligned} \tag{1.1}$$

with associated probability density functions, $g_R(r)$ and $g_\Theta(\theta)$. The distance travelled in the x-direction in time t, X_t, is given by $X_t = R_t H_t$, where $H_t = \cos(\Theta_t)$. Notice that R_t and H_t are independent, so that the probability density function is is $f_{X_t}(r,\theta) = g_{R_t}(r) f_{H_t}(\theta)$.

The pdf of H_t is given by considering

$$\begin{aligned} P(a \leq H \leq b) &= P(\cup_i \{\theta \in \operatorname{arccos}_k([a,b])\}) \\ &= \sum_k P(\theta \in \operatorname{arccos}_k([a,b])) && (1.2) \\ &= \sum_k \int_{\operatorname{arccos}_k([a,b]))} g_\Theta(u)\mathrm{du} && (1.3) \\ &= \sum_k \int_{\operatorname{arccos}_k([a,b]))} g_\Theta(u)\mathrm{du} && (1.4) \\ &= \sum_k \int_a^b g_\Theta(\operatorname{arccos}_k(s)) \left| \frac{d}{ds} \operatorname{arccos}_k(s) \right| \mathrm{ds} && (1.5) \\ &= \int_a^b \sum_k g_\Theta(\operatorname{arccos}_k(s)) \left| \frac{d}{ds} \operatorname{arccos}_k(s) \right| \mathrm{ds} && (1.6) \end{aligned}$$

where the sum is over the subscript k, such that for a given h, $\theta_k = \operatorname{arccos}_k(h)$ $\forall$ k. It follows immediately that the pdf for H_Θ is

$$f_H = \sum_i g_\Theta(\operatorname{arccos}_k(h)) \left| \frac{d}{ds} \operatorname{arccos}_k(h) \right| \tag{1.7}$$

Note that the set of solutions, T to $h = \cos(\theta)$, is given by:

$$T = \{\arccos(h) + 2\pi k, -\arccos(h) + 2\pi k \mid k \in \mathbb{Z}\} \tag{1.8}$$

where $\arccos(h)$ gives solutions in usual range of principle values $[0, 2\pi]$. We include solutions outside of the range $[-\pi, \pi]$ to allow for the possibility that a cell could turn through more than 2π in a time step (although, our aim was to record cell position at a frequency high enough to avoid that occurrence). Hence,

$$f_H(h) = \sum_k \frac{1}{(1-h^2)^{1/2}} (g_\Theta(\theta + 2\pi k) + g_\Theta(-\theta + 2\pi k) \tag{1.9}$$

Firstly note that f_H is integrable on [-1,1], which we will demonstrate below. Also note that as $t \to \infty$, $f_H(h) \to \frac{1}{\pi(1-h^2)^{1/2}}$, which is the distribution of $H = \cos(\Phi)$, where Φ is uniformly distributed. To see this note that:

$$\sum_{k=-\infty}^{\infty} (g_\Theta(\theta + 2\pi k) \quad + \quad g_\Theta(-\theta + 2\pi k)) =$$

$$\frac{1}{(2\pi\sigma_\theta^2 t)^{1/2}} \sum_{k=-\infty}^{\infty} e^{-\frac{(\theta_0+\theta+2\pi k)^2}{2\sigma_\theta^2 t}} \quad + \quad e^{-\frac{(\theta_0-\theta+2\pi k)^2}{2\sigma_\theta^2 t}} \tag{1.10}$$

If the summation is rewritten as $\sum_{k=-\infty}^{\infty} w(k)$ then as $t \to \infty$, $w(k+1) - w(k) \to 0$. Consider w, as a function of a continuous variable k', then as $t \to \infty$

$$\int_{k-1/2}^{k+1/2} w(k')\mathrm{dk}' \to \mathrm{w(k)} \int_{\mathrm{k}-1/2}^{\mathrm{k}+1/2} \mathrm{dk}' = \mathrm{w(k)} \tag{1.11}$$

Therefore, if $x = 2\pi k'$,

$$\sum_{k=-\infty}^{\infty} w(k) \to \frac{1}{2\pi} \int_{-\infty}^{\infty} w(x)\mathrm{dx} = 2\sigma_\theta \mathrm{t}^{1/2} \frac{(2\pi)^{1/2}}{2\pi} \tag{1.12}$$

Leading immediately to the required result. The pdf for X_t is easily found by multiplying the pdf for R_t (which is the standard Guassian pdf) by the pdf for H_t as above. However, this gives f_{X_t} as a function of r and θ. To find f_{X_t} as a

function of one variable, x (the distance traveled in the x-direction), consider

$$\begin{aligned} P(x < x' < x + \delta x) &= P(-1 < h < 1) P(r < r' < x/h + \delta x/h) && (1.13) \\ &= \int_{-1}^{1} \int_{x/h}^{x/h + \delta x/h} f_X(r', h) \mathrm{dhdr}' && (1.14) \\ &= \delta x \int_{-1}^{1} \frac{f_{X_t}(x/h, h)}{h} \mathrm{dh} && (1.15) \end{aligned}$$

Hence,

$$f_{X_t}(x) = \int_{-1}^{1} \frac{f_{X_t}(x/h, h)}{h} \mathrm{dh} \tag{1.16}$$

This gives the correct pdf, which we validated against stochastic simulations of our migration model.

2 Integration

The integration in equation 2.18 has to be done with care due to the singularities at $h = 0$ and $h = \pm 1$. Although, for $x >> 0$, we found the following approximation satisfactory:

$$f_{X_t}(x) \approx \frac{2}{N} \sum_{k=0}^{N} \frac{f_{X_t}(x/h_k, h_k)}{h_k} \tag{2.17}$$

where $h_k = -1 + 2k/N$. However, for $x \approx 0$ the singularities have to be removed. To do this the integral is split:

$$\begin{aligned} f_{X_t}(x) &= \int_{-1}^{1} \frac{f_{X_t}(x/h, h)}{h} \mathrm{dh} && (2.18) \\ &= \int_{-1}^{-\epsilon} \dots + \int_{-\epsilon}^{0} \dots + \int_{0}^{\epsilon} \dots + \int_{\epsilon}^{1} \dots && (2.19) \\ &= I_1 + I_2 + I_3 + I_4 && (2.20) \end{aligned}$$

For I_1 and I_4, the substitution $h = \cos\phi$ removes the singularity at $h = \pm 1$. For I_3 substitute $z = \ln(h)$, and for I_2 substitute $z = -\ln(-h)$, which both remove the singularity at zero. We take $\epsilon = 1/2$.

3 Comparison with Experimental Data

For general experimental data, tracks are recorded as a sequence of time points, whereas $f_{X_t}(x)$ is a pdf for the distance traveled in x for a set time. Hence, in order to analyze all the data available to us, we analyze the distance traveled in time Δt, which in this case we took to be the time between image frames (5 minutes). Equation 2.18 assumes that θ_0 is known and constant. Hence, to compare experimental data to this pdf, tracks are broken up into pairs of steps and a 'rigid-body' rotation is applied such that each step in the data set is initiated at $(r_0, \theta_0) = (0, 0)$, (see main text). To compare with the model, a histogram is plotted from the set of rotated steps with bin centers x_i, bin widths w_i, and resulting density $D(x_i)$. To quantify the model fit to the experimental data we compare the area of the histogram bars with the corresponding area under the pdf. If $f_{X_t}(x)$ is approximately linear in the range $x_i \in B_i = [x_i - w_i/2, x_i + w_i/2]$, then $\Delta s_i = (f_{X_t}(x_i) - D(x_i))^2$ is a good metric for the fit. However, in regions where $f_{X_t}(x)$ is highly non-linear (for example: $f'_{X_t}(x - w_i/2) >> 0$ and $f'_{X_t}(x + wi/2) << 0$), Δs_i is a poor metric of the fit. In these cases the area under the pdf, $A(x_i)$, is calculated (by computation of f_{X_t} at additional points) and $\Delta s_j = (A(x_j) - D(x_j)w_j)^2$ is used. The regions where this is deemed necessary are found by visual inspection of the experimental data. In the case of random cell migration we found this usually to be around $x = 0$.

4 Fitting Procedure

To find the best possible fit, we minimize $\Delta S = \sum_i \Delta s_i$. To minimize ΔS we used simulated annealing, a Monte-Carlo based method. Following an initialization of the problem, new parameter sets $\mathbf{P}_{k+1}$ are generated by adding gaussian noise to the existing set, $\mathbf{P}_k$. If $\Delta S_{k+1} < \Delta S_k$ then the new parameters are accepted. Otherwise they are only accepted with probability $e^{(\Delta S_k - \Delta S_{k+1})/T_k}$, where T_k is the 'temperature' of the simulation. The 'annealing' occurs due to a predefined cooling regime. Here we use geometric cooling so that $T_{k+1} = \alpha T_k$

where $\alpha < 1$. Given the stochastic nature of the procedure. a good fit is not guaranteed. Hence we repeat this fitting. The length of the fitting procedure is 4000 steps, the cooling lasts until step 3500, after which the selected parameters are recorded. This is repeated 200 times. The resulting parameters are plotted as histograms (fig. 4, supp. figs 3-4).

5 Confidence Interval Calculation

To find the best fit we sought to minimize ΔS, which is a function of a parameter set $\mathbf{p} = (\mu_{r_1}, \sigma_{r_1}, \sigma_{\theta_1}, \mu_{r_2}, \sigma_{r_2}, \sigma_{\theta_2})$, and the fractional weight of time spent in state 1, α, and of course is also dependent on the data D. Because they need to be treated slightly differently in what follows α and $\mathbf{p}$ are seperated. So, formally we sought

$$[\hat{\mathbf{p}}, \hat{\alpha}] = \underset{(\mathbf{p},\alpha)}{\arg\min}\, \Delta S(\mathbf{p}, \alpha; \mathbf{D}) \tag{5.21}$$

so $\hat{\mathbf{p}}$ and $\hat{\alpha}$ are the values of $\mathbf{p}$ and α which minimize ΔS. To profile our confidence in the estimates for $\hat{\alpha}$ we simply calculated

$$\Delta S(\hat{\mathbf{p}}, \alpha),\ \ \alpha \in [0, 1] \tag{5.22}$$

Then we can capture values of α which satisfy $\Delta S(\hat{\mathbf{p}}, \alpha) \leq \Delta S(\hat{\mathbf{p}}, \hat{\alpha}) + \epsilon$, where ϵ is some threshold for 'accepting' a parameter value to be defined and determined below.

To treat the other parameters we do something similar, but add an additional step. For each element in the parameter set $\mathbf{p}$ we fix it at a given value and re-optimize the other elements in $\mathbf{p}$. Define $\mathbf{q_i} = \mathbf{p}/p_i$. Then we seek

$$\Delta S(\hat{\mathbf{q}}_\mathbf{i}, p_i, \hat{\alpha}) = \min_{(\mathbf{q_i},\hat{\alpha})} \Delta S(\mathbf{q_i}, p_i, \hat{\alpha}; \mathbf{D}) \tag{5.23}$$

Then, similarly to above, we can look for the values of p_i which satisfy $\Delta S(\hat{\mathbf{q}}_\mathbf{i}, p_i, \hat{\alpha}) \leq \Delta S(\hat{\mathbf{p}}, \hat{\alpha}) + \epsilon$.

Note that we treat α and $\mathbf{p}$ distinctly because when $\alpha = 0$, (or $\alpha = 1$) the model results are independent of $(\mu_{r_1}, \sigma_{r_1}, \sigma_{\theta_1})$ (or $(\mu_{r_2}, \sigma_{r_2}, \sigma_{\theta_2})$), therefore if you do this simultaneously you introduce degeneracy in this approach. In some circumstances this may be desirable, but in this case we found fixing α or $\mathbf{p}$ to be more informative[1] However, in what follows, any statements we make about the confidence in the parameter values should be interpreted as conditional on the best fit values $\hat{\alpha}$ in the case of the members of $\mathbf{p}$, and $\hat{\mathbf{p}}$ in the case of α.

To set the threshold ϵ we want to include parameter values that we could conclude were the best fit if we repeat the experiment. Therefore we need an estimate of the variability of the data. To do this we resample the data using bootstrap sampling and score the new sample against the actual dataset. Since our data is a collection of steps the cell took at a given time-step, we resample at this level. However, it is also possible to keep entire cell tracks intact in the resampling process by simply redrawing what cells are included in the new dataset. In either case we build a distribution for values of ΔS which we could plausibly see upon resampling (or, to rephrase, if we had a perfect model that exactly matches the full data set, how would that model score against repeats of the experiment?). In either case (resampling steps, or whole tracks) we select ϵ such that 80% of the observed values fall below $\Delta S(\hat{\mathbf{p}}, \hat{\alpha}) + \epsilon$. Then the parameter values that are predicted to lead to model fits in this range define the 10th to 90th confidence interval in each parameter.

[1]The reason why this is more informative is that otherwise this approach does not take in to account how the relationship between the parameters is constrained. For example, suppose our model is simply $f = \alpha p + (1 - \alpha)q$, and that our data is $1 \leq f \leq 10$ then if $\alpha \in [0, 1]$ our bounds for both p and q are [1,10] but these ranges do not convey that the model constrains the possible combinations for α, p, and q, even though for any one parameter the range is not constrained.
